# *N*-{Bis[meth­yl(phen­yl)amino]phosphor­yl}-2,2,2-trichloro­acetamide

**DOI:** 10.1107/S1600536809042391

**Published:** 2009-10-23

**Authors:** Kateryna O. Znovjyak, Vladimir A. Ovchynnikov, Tetyana Yu. Sliva, Svitlana V. Shishkina, Vladimir M. Amirkhanov

**Affiliations:** a Department of Chemistry, Kyiv National Taras Shevchenko University, Volodymyrska str. 64, 01033 Kyiv, Ukraine; b STC "Institute for Single Crystals", National Academy of Science of Ukraine, Lenina ave. 60, 61001, Khar’kov, Ukraine

## Abstract

In the asymmetric unit of the crystal structure of the title compound, C_16_H_17_Cl_3_N_3_O_2_P, there are two crystallograph­ically independent mol­ecules, which form dimers *via* N—H⋯O hydrogen bonding between the N—H group and the P=O group. In the mol­ecular structure, the phosphoryl group is *anti* to the carbonyl group. The two benzene rings are oriented at dihedral angles of 54.3 (2) and 49.7 (2)° in the two independent mol­ecules.

## Related literature

For background to the chemistry of phospho­rus-containing systems, see: Helm *et al.* (1999 ▶); Katti *et al.* (1991 ▶). For the biological and pharmacological properties of carbacyl­amido­phosphate derivatives, see: Jaroslav & Swetdloff (1985 ▶). For structural and conformational studies of related mol­ecules, see: Gholivand *et al.* (2008*a* ▶,*b* ▶); Gubina *et al.* (1999 ▶); Rebrova *et al.* (1982 ▶). For the coordination properties of carbacyl­amido­phosphates, see: Oczko *et al.* (2003 ▶); Amirkhanov *et al.* (1997 ▶); Trush *et al.* (2003 ▶); Gubina *et al.* (2002 ▶). For details of the synthesis, see Kirsanov & Derkach (1956 ▶).
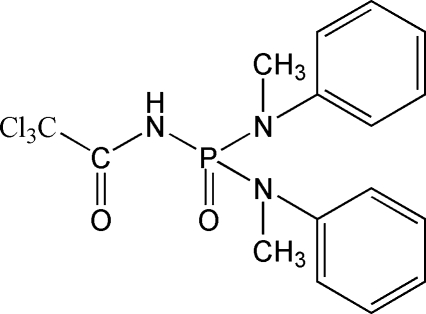


## Experimental

### 

#### Crystal data

C_16_H_17_Cl_3_N_3_O_2_P
*M* *_r_* = 420.65
Monoclinic, 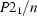

*a* = 12.8226 (10) Å
*b* = 19.5161 (15) Å
*c* = 15.1132 (12) Å
β = 93.345 (6)°
*V* = 3775.6 (5) Å^3^
*Z* = 8
Mo *K*α radiation
μ = 0.59 mm^−1^
*T* = 293 K
0.40 × 0.20 × 0.05 mm

#### Data collection

Oxford Diffraction Xcalibur3 diffractometer
Absorption correction: multi-scan (*CrysAlis RED*; Oxford Diffraction, 2006 ▶) *T*_min_ = 0.800, *T*_max_ = 0.971
37663 measured reflections
8168 independent reflections
6768 reflections with *I* > 2σ(*I*)
*R*_int_ = 0.086

#### Refinement

*R*[*F* ^2^ > 2σ(*F* ^2^)] = 0.094
*wR*(*F* ^2^) = 0.174
*S* = 1.16
8168 reflections
451 parameters
H-atom parameters constrained
Δρ_max_ = 0.50 e Å^−3^
Δρ_min_ = −0.50 e Å^−3^

### 

Data collection: *CrysAlis CCD* (Oxford Diffraction, 2006 ▶); cell refinement: *CrysAlis RED* (Oxford Diffraction, 2006 ▶); data reduction: *CrysAlis RED*; program(s) used to solve structure: *SHELXS97* (Sheldrick, 2008 ▶); program(s) used to refine structure: *SHELXL97* (Sheldrick, 2008 ▶); molecular graphics: *ORTEP-3 for Windows* (Farrugia, 1997 ▶); software used to prepare material for publication: *WinGX* (Farrugia, 1999 ▶).

## Supplementary Material

Crystal structure: contains datablocks I, global. DOI: 10.1107/S1600536809042391/xu2626sup1.cif

Structure factors: contains datablocks I. DOI: 10.1107/S1600536809042391/xu2626Isup2.hkl

Additional supplementary materials:  crystallographic information; 3D view; checkCIF report

## Figures and Tables

**Table 1:** Hydrogen-bond geometry (Å, °)

*D*—H⋯*A*	*D*—H	H⋯*A*	*D*⋯*A*	*D*—H⋯*A*
N11—H11*B*⋯O21	0.86	1.91	2.741 (5)	161
N21—H21*B*⋯O11	0.86	1.93	2.752 (5)	159

**Fig. 1.**
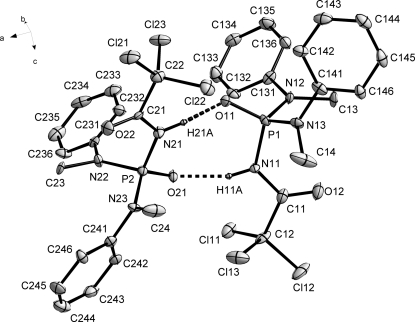
A view of hydrogen bonding between the two independent molecules in the unit cell of the title compound with atom numbering scheme. Displacement ellipsoids are shown at 50% probability level. Hydrogen atoms not involved in hydrogen bonding are omitted for clarity.

**Fig. 2.**
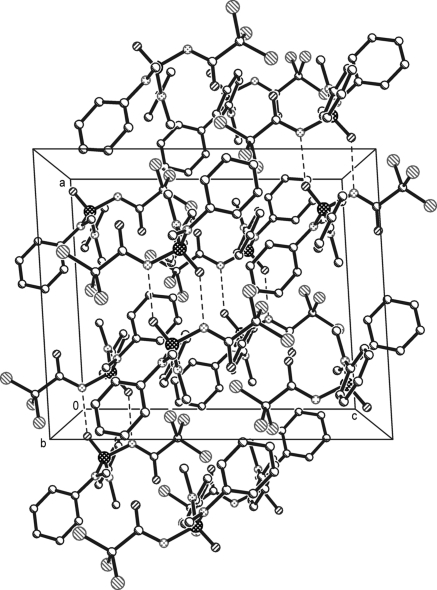
A projection of packing diagram of the title compound along the y-direction.

## supplementary crystallographic information

### Comment

The intensive development of the chemistry of the phosphorus containing systems during the last decades has given rise to synthesis of many compounds bonded by phosphorus and nitrogen atoms (Helm *et al.*, 1999; Katti *et al.*, 1991). Carbacylamidophosphates, which have CONHPO in molecular core unit, have attracted attention because of their using in pharmacology, as insecticides, pesticides and ureas inhibitor (Jaroslav *et al.*, 1985). These types of compounds are used as ligands, particularly for lanthanides (Oczko *et al.*, 2003), actinides (Amirkhanov *et al.*, 1997) and 3d-metals (Trush *et al. *, 2003; Gubina *et al.*, 2002). Thus to date the syntheses and structures of carbacylamidophosphate compounds have been of increased interest (Gholivand *et al.*, 2008a). This paper is devoted to the crystal structure of *N*-{bis[methyl(phenyl)amino]phosphoryl}-2,2,2-trichloroacetamide (HL).

The title compound contains two crystallographically independent molecules in the asymmetric unit of the unit cell, which aggregate into the non-centrosymmetric dimers (HL)_2_ formed by the hydrogen bonds of N—H···OP (Figs. 1 and 2, Table 1). In the crystal structure of the HL the phosphoryl and carbonyl groups are in an *anti*-position to each other as in the most of the carbacylamidophosphates (Gubina *et al.*, 1999). The bond distances P(1)O(11) and P(2)O(21) are typical for compounds with amide substituents close to phosphorus atoms (1.465–1.482 Å) (Rebrova *et al.*, 1982). The values of CO and C—N bonds lie in the ranges 1.193 (8) Å, 1.207 (8) Å and 1.365 (9) Å, 1.362 (8) Å, respectively and close to the corresponding values of the carbacylamidophosphates (Gholivand *et al.*, 2008b). The P(1)—N(11) (1.684 (5) Å) and P(2)—N(21) (1.692 (5) Å) distances of HL are longer on average by 0.05 Å than P—N bond distances between amide substituents and phosphorus atoms (P(1)—N(12), P(1)—N(13), P(2)—N(22), P(2)—N(23)) which fall in the range 1.634 (4)–1.649 (4) Å.

The phosphorus atoms of HL have distorted tetrahedral configuration where the values of O—P—N angles have the highest deviation from the 109.28°. Inspection of the O(11)—P(1)—N(11) and O(21)—P(2)—N(21) angles indicates that these angles are less than tetrahedral one (105.9 (2)° and 105.4 (2)°, respectively), while others O—P—N angles indicate higher values (111.2 (2)°-119.0 (2)°), that may be explained by the repulsion of amide substituents and PO group.

### Experimental

The dichloranhydride of trichloroacetylamidophosphoric acid was prepared according to the method reported by Kirsanov (Kirsanov *et al.*, 1956).

The dioxane solution (100 ml) of methylaniline (21.4 g, 0.2 mol) and triethylamine (20.2 g, 0.2 mol) was placed in a three-neck round-bottomed flask and cooled by ice to 268 K. Then the dioxane solution (400 ml) of dichloranhydride of trichloroacetylamidophosphoric acid (27.9 g, 0.1 mol) was added dropwise under vigorous stirring. The temperature was not allowed to rise above 278 K. The stirring was continued for 1 h. The formed precipitate of N(C_2_H_5_)_3_HCl was filtered off and the filtrate evaporated. The oily precipitate of *N*-{bis[methyl(phenyl)amino]phosphoryl}-2,2,2-trichloroacetamide was isolated and recrystallized from the 2-propanol as white crystalline powder. The colourless crystals of the HL were obtained by slow evaporation of the mother liquor, washed with cool 2-propanol (10 ml) and finally dried in air (yield 85%).

### Refinement

All H atoms were placed at calculated positions and treated as riding on their parent atoms [C—H = 0.93 and 0.96 Å, and *U*_iso_(H) = 1.2 and 1.5*U*_eq_(C), N—H = 0.86 Å and *U*_iso_(H) = 1.2*U*_eq_(N)].

### Crystal data

**Table:**

C_16_H_17_Cl_3_N_3_O_2_P	*F*(000) = 1728
*M**_r_* = 420.65	*D*_x_ = 1.480 Mg m^−^^3^
Monoclinic, *P*2_1_/*n*	Mo *K*α radiation, λ = 0.71073 Å
Hall symbol: -P 2yn	Cell parameters from 51957 reflections
*a* = 12.8226 (10) Å	θ = 2.6–32.2°
*b* = 19.5161 (15) Å	µ = 0.59 mm^−^^1^
*c* = 15.1132 (12) Å	*T* = 293 K
β = 93.345 (6)°	Plate, colourless
*V* = 3775.6 (5) Å^3^	0.40 × 0.20 × 0.05 mm
*Z* = 8	

### Data collection

**Table:**

Oxford Diffraction Xcalibur3 diffractometer	8168 independent reflections
Radiation source: Enhance (Mo) X-ray Source	6768 reflections with *I* > 2σ(*I*)
graphite	*R*_int_ = 0.086
Detector resolution: 16.1827 pixels mm^-1^	θ_max_ = 27.5°, θ_min_ = 2.6°
ω scans	*h* = −16→16
Absorption correction: multi-scan (*CrysAlis RED*; Oxford Diffraction, 2006)	*k* = −25→25
*T*_min_ = 0.800, *T*_max_ = 0.971	*l* = −19→19
37663 measured reflections	

### Refinement

**Table:**

Refinement on *F*^2^	Primary atom site location: structure-invariant direct methods
Least-squares matrix: full	Secondary atom site location: difference Fourier map
*R*[*F*^2^ > 2σ(*F*^2^)] = 0.094	Hydrogen site location: inferred from neighbouring sites
*wR*(*F*^2^) = 0.174	H-atom parameters constrained
*S* = 1.16	*w* = 1/[σ^2^(*F*_o_^2^) + (0.0501*P*)^2^ + 9.3908*P*] where *P* = (*F*_o_^2^ + 2*F*_c_^2^)/3
8168 reflections	(Δ/σ)_max_ = 0.002
451 parameters	Δρ_max_ = 0.50 e Å^−^^3^
0 restraints	Δρ_min_ = −0.50 e Å^−^^3^

### Special details

**Table:**

Experimental. ^1^H NMR, 400.13 MHz, (DMSO-*d_6_*): 3.06, 3.08 (d, 6H, CH_3_), 7.3 (m, 10H, C_6_H_5_), 10.28 (s, 1H, NH). ^31^P NMR, 162.1 MHz, (DMSO-*d_6_*): 5.46 (*m*). IR (KBr pellet, cm^1^): 3040 (s, N—H), 2850, 1730(s, CO), 1600 (s, CC), 1470 (s, C—N), 1280, 1245, 1210(s, PO), 1090, 1039, 930, 885, 815, 700, 685 (s, C—Cl).
Geometry. All e.s.d.'s (except the e.s.d. in the dihedral angle between two l.s. planes) are estimated using the full covariance matrix. The cell e.s.d.'s are taken into account individually in the estimation of e.s.d.'s in distances, angles and torsion angles; correlations between e.s.d.'s in cell parameters are only used when they are defined by crystal symmetry. An approximate (isotropic) treatment of cell e.s.d.'s is used for estimating e.s.d.'s involving l.s. planes.
Refinement. Refinement of *F*^2^ against ALL reflections. The weighted *R*-factor *wR* and goodness of fit *S* are based on *F*^2^, conventional *R*-factors *R* are based on *F*, with *F* set to zero for negative *F*^2^. The threshold expression of *F*^2^ > σ(*F*^2^) is used only for calculating *R*-factors(gt) *etc*. and is not relevant to the choice of reflections for refinement. *R*-factors based on *F*^2^ are statistically about twice as large as those based on *F*, and *R*- factors based on ALL data will be even larger.

### Fractional atomic coordinates and isotropic or equivalent isotropic displacement parameters (Å^2^)

**Table:**

	*x*	*y*	*z*	*U*_iso_*/*U*_eq_	
P1	0.33568 (8)	0.18559 (6)	0.59326 (7)	0.0137 (2)	
Cl11	0.48523 (10)	0.22113 (6)	0.87366 (7)	0.0261 (3)	
Cl12	0.34981 (12)	0.11928 (8)	0.94348 (8)	0.0370 (3)	
Cl13	0.51399 (12)	0.07733 (7)	0.83310 (9)	0.0365 (3)	
O11	0.4195 (2)	0.20901 (16)	0.53685 (19)	0.0171 (7)	
N12	0.2449 (3)	0.24477 (19)	0.6008 (3)	0.0168 (8)	
N13	0.2727 (3)	0.1149 (2)	0.5658 (2)	0.0183 (8)	
N11	0.3948 (3)	0.1685 (2)	0.6933 (2)	0.0183 (8)	
H11B	0.4617	0.1718	0.6983	0.022*	
C131	0.2676 (3)	0.3157 (2)	0.5929 (3)	0.0154 (9)	
C12	0.4208 (4)	0.1426 (2)	0.8517 (3)	0.0197 (10)	
C141	0.1944 (3)	0.1152 (2)	0.4930 (3)	0.0179 (9)	
C142	0.2132 (4)	0.1471 (2)	0.4134 (3)	0.0209 (10)	
H14A	0.2771	0.1681	0.4056	0.025*	
C132	0.3617 (3)	0.3435 (2)	0.6318 (3)	0.0185 (10)	
H13A	0.4117	0.3149	0.6595	0.022*	
O12	0.2530 (3)	0.1401 (2)	0.7707 (2)	0.0311 (9)	
C11	0.3452 (4)	0.1500 (2)	0.7667 (3)	0.0195 (10)	
C146	0.0988 (4)	0.0838 (2)	0.5038 (3)	0.0216 (10)	
H14B	0.0869	0.0622	0.5571	0.026*	
C13	0.1359 (3)	0.2280 (2)	0.6205 (3)	0.0192 (10)	
H13B	0.0955	0.2693	0.6215	0.029*	
H13C	0.1355	0.2058	0.6771	0.029*	
H13D	0.1062	0.1979	0.5755	0.029*	
C133	0.3785 (4)	0.4132 (3)	0.6281 (3)	0.0247 (11)	
H13E	0.4402	0.4315	0.6535	0.030*	
C145	0.0208 (4)	0.0844 (2)	0.4363 (3)	0.0254 (11)	
H14C	−0.0435	0.0639	0.4442	0.030*	
C144	0.0408 (4)	0.1166 (3)	0.3554 (3)	0.0282 (12)	
H14D	−0.0101	0.1169	0.3089	0.034*	
C136	0.1951 (4)	0.3596 (3)	0.5512 (3)	0.0247 (11)	
H13F	0.1332	0.3418	0.5255	0.030*	
C134	0.3039 (4)	0.4568 (3)	0.5869 (3)	0.0280 (11)	
H13G	0.3150	0.5039	0.5860	0.034*	
C135	0.2133 (4)	0.4290 (3)	0.5474 (3)	0.0258 (11)	
H13H	0.1644	0.4574	0.5180	0.031*	
C143	0.1355 (4)	0.1473 (3)	0.3458 (3)	0.0254 (11)	
H14E	0.1480	0.1689	0.2926	0.030*	
C14	0.2943 (6)	0.0485 (3)	0.6085 (4)	0.0474 (18)	
H14F	0.2489	0.0143	0.5817	0.071*	
H14G	0.2826	0.0518	0.6705	0.071*	
H14H	0.3657	0.0359	0.6013	0.071*	
P2	0.68252 (8)	0.17932 (6)	0.63323 (7)	0.0135 (2)	
Cl21	0.66403 (11)	0.10956 (7)	0.28388 (8)	0.0311 (3)	
Cl22	0.48977 (10)	0.08272 (7)	0.39052 (8)	0.0278 (3)	
Cl23	0.54923 (11)	0.22269 (6)	0.35201 (8)	0.0294 (3)	
O21	0.6002 (2)	0.20639 (17)	0.68822 (19)	0.0179 (7)	
N22	0.7759 (3)	0.23606 (19)	0.6228 (2)	0.0149 (8)	
N23	0.7424 (3)	0.1085 (2)	0.6634 (2)	0.0163 (8)	
N21	0.6213 (3)	0.1617 (2)	0.5331 (2)	0.0160 (8)	
H21B	0.5546	0.1665	0.5281	0.019*	
O22	0.7598 (2)	0.12398 (19)	0.4579 (2)	0.0246 (8)	
C241	0.8228 (3)	0.1087 (2)	0.7325 (3)	0.0174 (9)	
C22	0.5976 (4)	0.1391 (2)	0.3743 (3)	0.0218 (10)	
C21	0.6696 (3)	0.1405 (2)	0.4607 (3)	0.0157 (9)	
C246	0.9151 (3)	0.0725 (2)	0.7220 (3)	0.0197 (10)	
H24A	0.9224	0.0466	0.6710	0.024*	
C23	0.8826 (3)	0.2165 (2)	0.6004 (3)	0.0186 (9)	
H23A	0.9252	0.2569	0.5981	0.028*	
H23B	0.8797	0.1941	0.5438	0.028*	
H23C	0.9121	0.1859	0.6449	0.028*	
C231	0.7582 (3)	0.3072 (2)	0.6305 (3)	0.0158 (9)	
C232	0.6668 (4)	0.3381 (3)	0.5922 (3)	0.0202 (10)	
H23D	0.6148	0.3111	0.5645	0.024*	
C243	0.8935 (4)	0.1479 (3)	0.8756 (3)	0.0286 (12)	
H24B	0.8859	0.1729	0.9272	0.034*	
C242	0.8124 (4)	0.1458 (2)	0.8104 (3)	0.0186 (9)	
H24C	0.7507	0.1694	0.8188	0.022*	
C236	0.8343 (4)	0.3489 (2)	0.6720 (3)	0.0200 (10)	
H23E	0.8945	0.3293	0.6984	0.024*	
C235	0.8212 (4)	0.4195 (3)	0.6744 (3)	0.0292 (12)	
H23F	0.8730	0.4466	0.7023	0.035*	
C234	0.7323 (4)	0.4501 (3)	0.6359 (3)	0.0282 (12)	
H23G	0.7247	0.4975	0.6369	0.034*	
C233	0.6546 (4)	0.4089 (3)	0.5958 (3)	0.0256 (11)	
H23H	0.5938	0.4289	0.5712	0.031*	
C245	0.9958 (4)	0.0747 (3)	0.7867 (4)	0.0292 (12)	
H24D	1.0572	0.0508	0.7787	0.035*	
C244	0.9856 (4)	0.1127 (3)	0.8639 (3)	0.0283 (12)	
H24E	1.0401	0.1143	0.9071	0.034*	
C24	0.7110 (5)	0.0406 (3)	0.6298 (4)	0.0377 (15)	
H24F	0.7568	0.0065	0.6562	0.057*	
H24G	0.7150	0.0396	0.5665	0.057*	
H24H	0.6405	0.0313	0.6445	0.057*	

### Atomic displacement parameters (Å^2^)

**Table:**

	*U*^11^	*U*^22^	*U*^33^	*U*^12^	*U*^13^	*U*^23^
P1	0.0101 (5)	0.0170 (6)	0.0141 (5)	0.0013 (4)	0.0009 (4)	−0.0008 (4)
Cl11	0.0343 (6)	0.0281 (6)	0.0160 (6)	−0.0131 (5)	0.0028 (5)	−0.0025 (5)
Cl12	0.0538 (9)	0.0416 (8)	0.0167 (6)	−0.0202 (7)	0.0116 (6)	0.0007 (6)
Cl13	0.0501 (8)	0.0352 (7)	0.0235 (6)	0.0177 (7)	−0.0046 (6)	0.0018 (6)
O11	0.0131 (14)	0.0230 (17)	0.0154 (15)	0.0037 (13)	0.0022 (12)	0.0021 (13)
N12	0.0096 (17)	0.0166 (19)	0.024 (2)	−0.0009 (15)	0.0042 (15)	−0.0009 (16)
N13	0.0184 (19)	0.018 (2)	0.0180 (19)	0.0055 (16)	0.0004 (15)	−0.0008 (16)
N11	0.0138 (18)	0.027 (2)	0.0139 (18)	−0.0017 (16)	0.0012 (15)	0.0016 (16)
C131	0.022 (2)	0.013 (2)	0.012 (2)	−0.0011 (18)	0.0037 (17)	−0.0048 (17)
C12	0.028 (2)	0.020 (2)	0.011 (2)	−0.006 (2)	0.0058 (18)	−0.0021 (18)
C141	0.017 (2)	0.021 (2)	0.015 (2)	0.0052 (18)	−0.0025 (17)	−0.0057 (18)
C142	0.022 (2)	0.020 (2)	0.020 (2)	0.0019 (19)	−0.0003 (19)	−0.0003 (19)
C132	0.016 (2)	0.019 (2)	0.020 (2)	0.0038 (18)	−0.0027 (18)	−0.0049 (19)
O12	0.0237 (18)	0.045 (2)	0.0250 (19)	−0.0120 (17)	0.0089 (15)	0.0000 (17)
C11	0.018 (2)	0.023 (2)	0.018 (2)	−0.0042 (19)	0.0045 (18)	−0.0016 (19)
C146	0.022 (2)	0.020 (2)	0.023 (2)	−0.0019 (19)	0.0037 (19)	−0.001 (2)
C13	0.011 (2)	0.020 (2)	0.028 (2)	0.0007 (18)	0.0060 (18)	−0.004 (2)
C133	0.022 (2)	0.023 (3)	0.028 (3)	−0.003 (2)	−0.003 (2)	−0.004 (2)
C145	0.025 (3)	0.016 (2)	0.035 (3)	−0.001 (2)	−0.006 (2)	−0.005 (2)
C144	0.029 (3)	0.026 (3)	0.029 (3)	0.009 (2)	−0.010 (2)	−0.010 (2)
C136	0.020 (2)	0.030 (3)	0.023 (3)	0.002 (2)	−0.004 (2)	0.000 (2)
C134	0.035 (3)	0.023 (3)	0.026 (3)	−0.004 (2)	0.002 (2)	0.001 (2)
C135	0.029 (3)	0.018 (2)	0.030 (3)	0.005 (2)	−0.004 (2)	0.004 (2)
C143	0.029 (3)	0.031 (3)	0.016 (2)	0.007 (2)	0.001 (2)	0.001 (2)
C14	0.070 (4)	0.018 (3)	0.050 (4)	0.008 (3)	−0.036 (3)	−0.003 (3)
P2	0.0119 (5)	0.0167 (6)	0.0121 (5)	−0.0011 (4)	0.0019 (4)	−0.0004 (4)
Cl21	0.0445 (8)	0.0346 (7)	0.0148 (6)	0.0109 (6)	0.0077 (5)	−0.0020 (5)
Cl22	0.0254 (6)	0.0321 (6)	0.0255 (6)	−0.0047 (5)	−0.0026 (5)	−0.0060 (6)
Cl23	0.0440 (8)	0.0250 (6)	0.0190 (6)	0.0131 (6)	−0.0002 (5)	0.0036 (5)
O21	0.0132 (15)	0.0270 (18)	0.0137 (15)	−0.0029 (13)	0.0026 (12)	−0.0028 (13)
N22	0.0092 (16)	0.0137 (18)	0.022 (2)	0.0001 (14)	0.0035 (14)	0.0001 (15)
N23	0.0163 (18)	0.0181 (19)	0.0143 (18)	0.0005 (15)	−0.0014 (15)	−0.0018 (15)
N21	0.0102 (17)	0.027 (2)	0.0108 (18)	−0.0024 (15)	0.0007 (14)	−0.0025 (15)
O22	0.0184 (17)	0.040 (2)	0.0163 (16)	0.0067 (15)	0.0070 (13)	−0.0031 (15)
C241	0.018 (2)	0.012 (2)	0.022 (2)	−0.0018 (18)	0.0010 (18)	0.0049 (18)
C22	0.024 (2)	0.021 (2)	0.019 (2)	0.002 (2)	0.0006 (19)	−0.0039 (19)
C21	0.015 (2)	0.019 (2)	0.013 (2)	−0.0021 (18)	0.0012 (17)	0.0024 (17)
C246	0.021 (2)	0.016 (2)	0.022 (2)	0.0041 (19)	−0.0019 (19)	−0.0015 (19)
C23	0.012 (2)	0.019 (2)	0.025 (2)	−0.0022 (18)	0.0021 (18)	0.0058 (19)
C231	0.016 (2)	0.020 (2)	0.013 (2)	0.0018 (18)	0.0052 (17)	0.0018 (17)
C232	0.016 (2)	0.027 (3)	0.018 (2)	0.0048 (19)	0.0019 (18)	0.0055 (19)
C243	0.034 (3)	0.030 (3)	0.020 (3)	−0.007 (2)	−0.008 (2)	0.000 (2)
C242	0.021 (2)	0.017 (2)	0.018 (2)	0.0008 (19)	0.0017 (18)	0.0022 (18)
C236	0.026 (2)	0.021 (2)	0.013 (2)	−0.002 (2)	−0.0007 (18)	0.0002 (18)
C235	0.045 (3)	0.019 (3)	0.023 (3)	−0.009 (2)	0.002 (2)	−0.007 (2)
C234	0.050 (3)	0.015 (2)	0.020 (2)	0.004 (2)	0.008 (2)	0.003 (2)
C233	0.035 (3)	0.024 (3)	0.018 (2)	0.010 (2)	0.006 (2)	0.004 (2)
C245	0.025 (3)	0.027 (3)	0.035 (3)	0.008 (2)	−0.002 (2)	0.009 (2)
C244	0.030 (3)	0.028 (3)	0.026 (3)	−0.004 (2)	−0.011 (2)	0.003 (2)
C24	0.058 (4)	0.019 (3)	0.033 (3)	−0.006 (3)	−0.023 (3)	0.000 (2)

### Geometric parameters (Å, °)

**Table:**

P1—O11	1.483 (3)	P2—O21	1.478 (3)
P1—N13	1.639 (4)	P2—N23	1.634 (4)
P1—N12	1.649 (4)	P2—N22	1.645 (4)
P1—N11	1.684 (4)	P2—N21	1.698 (4)
Cl11—C12	1.763 (5)	Cl21—C22	1.749 (5)
Cl12—C12	1.763 (5)	Cl22—C22	1.795 (5)
Cl13—C12	1.780 (5)	Cl23—C22	1.771 (5)
N12—C131	1.421 (6)	N22—C231	1.413 (6)
N12—C13	1.482 (5)	N22—C23	1.478 (5)
N13—C141	1.444 (6)	N23—C241	1.424 (6)
N13—C14	1.469 (6)	N23—C24	1.466 (6)
N11—C11	1.359 (6)	N21—C21	1.353 (5)
N11—H11B	0.8600	N21—H21B	0.8600
C131—C136	1.388 (6)	O22—C21	1.203 (5)
C131—C132	1.419 (6)	C241—C242	1.396 (6)
C12—C11	1.570 (7)	C241—C246	1.396 (6)
C141—C142	1.388 (6)	C22—C21	1.555 (6)
C141—C146	1.388 (6)	C246—C245	1.382 (7)
C142—C143	1.385 (7)	C246—H24A	0.9300
C142—H14A	0.9300	C23—H23A	0.9600
C132—C133	1.379 (7)	C23—H23B	0.9600
C132—H13A	0.9300	C23—H23C	0.9600
O12—C11	1.203 (6)	C231—C236	1.391 (6)
C146—C145	1.386 (7)	C231—C232	1.412 (6)
C146—H14B	0.9300	C232—C233	1.393 (7)
C13—H13B	0.9600	C232—H23D	0.9300
C13—H13C	0.9600	C243—C244	1.387 (8)
C13—H13D	0.9600	C243—C242	1.391 (6)
C133—C134	1.399 (7)	C243—H24B	0.9300
C133—H13E	0.9300	C242—H24C	0.9300
C145—C144	1.412 (8)	C236—C235	1.389 (7)
C145—H14C	0.9300	C236—H23E	0.9300
C144—C143	1.371 (7)	C235—C234	1.385 (8)
C144—H14D	0.9300	C235—H23F	0.9300
C136—C135	1.376 (7)	C234—C233	1.392 (7)
C136—H13F	0.9300	C234—H23G	0.9300
C134—C135	1.386 (7)	C233—H23H	0.9300
C134—H13G	0.9300	C245—C244	1.395 (7)
C135—H13H	0.9300	C245—H24D	0.9300
C143—H14E	0.9300	C244—H24E	0.9300
C14—H14F	0.9600	C24—H24F	0.9600
C14—H14G	0.9600	C24—H24G	0.9600
C14—H14H	0.9600	C24—H24H	0.9600
			
O11—P1—N13	118.48 (19)	O21—P2—N23	119.01 (19)
O11—P1—N12	111.25 (19)	O21—P2—N22	111.15 (19)
N13—P1—N12	105.49 (19)	N23—P2—N22	105.29 (19)
O11—P1—N11	105.88 (18)	O21—P2—N21	105.39 (18)
N13—P1—N11	104.5 (2)	N23—P2—N21	105.18 (19)
N12—P1—N11	111.0 (2)	N22—P2—N21	110.60 (19)
C131—N12—C13	115.6 (4)	C231—N22—C23	115.3 (3)
C131—N12—P1	121.8 (3)	C231—N22—P2	122.2 (3)
C13—N12—P1	122.5 (3)	C23—N22—P2	122.5 (3)
C141—N13—C14	116.3 (4)	C241—N23—C24	115.4 (4)
C141—N13—P1	120.0 (3)	C241—N23—P2	120.7 (3)
C14—N13—P1	123.6 (3)	C24—N23—P2	123.5 (3)
C11—N11—P1	125.3 (3)	C21—N21—P2	124.9 (3)
C11—N11—H11B	117.3	C21—N21—H21B	117.5
P1—N11—H11B	117.3	P2—N21—H21B	117.5
C136—C131—C132	118.8 (4)	C242—C241—C246	118.9 (4)
C136—C131—N12	120.3 (4)	C242—C241—N23	121.3 (4)
C132—C131—N12	120.8 (4)	C246—C241—N23	119.8 (4)
C11—C12—Cl11	109.5 (3)	C21—C22—Cl21	111.7 (3)
C11—C12—Cl12	110.2 (3)	C21—C22—Cl23	109.1 (3)
Cl11—C12—Cl12	109.5 (2)	Cl21—C22—Cl23	109.6 (3)
C11—C12—Cl13	109.0 (3)	C21—C22—Cl22	108.7 (3)
Cl11—C12—Cl13	109.9 (3)	Cl21—C22—Cl22	108.6 (3)
Cl12—C12—Cl13	108.7 (3)	Cl23—C22—Cl22	109.0 (3)
C142—C141—C146	119.8 (4)	O22—C21—N21	126.7 (4)
C142—C141—N13	120.9 (4)	O22—C21—C22	119.3 (4)
C146—C141—N13	119.2 (4)	N21—C21—C22	114.0 (4)
C143—C142—C141	119.2 (5)	C245—C246—C241	120.5 (5)
C143—C142—H14A	120.4	C245—C246—H24A	119.8
C141—C142—H14A	120.4	C241—C246—H24A	119.8
C133—C132—C131	119.5 (4)	N22—C23—H23A	109.5
C133—C132—H13A	120.3	N22—C23—H23B	109.5
C131—C132—H13A	120.3	H23A—C23—H23B	109.5
O12—C11—N11	126.2 (4)	N22—C23—H23C	109.5
O12—C11—C12	120.4 (4)	H23A—C23—H23C	109.5
N11—C11—C12	113.4 (4)	H23B—C23—H23C	109.5
C145—C146—C141	121.0 (5)	C236—C231—N22	120.0 (4)
C145—C146—H14B	119.5	C236—C231—C232	118.6 (4)
C141—C146—H14B	119.5	N22—C231—C232	121.2 (4)
N12—C13—H13B	109.5	C233—C232—C231	120.0 (5)
N12—C13—H13C	109.5	C233—C232—H23D	120.0
H13B—C13—H13C	109.5	C231—C232—H23D	120.0
N12—C13—H13D	109.5	C244—C243—C242	120.1 (5)
H13B—C13—H13D	109.5	C244—C243—H24B	119.9
H13C—C13—H13D	109.5	C242—C243—H24B	119.9
C132—C133—C134	120.9 (5)	C243—C242—C241	120.5 (5)
C132—C133—H13E	119.5	C243—C242—H24C	119.7
C134—C133—H13E	119.5	C241—C242—H24C	119.7
C146—C145—C144	118.8 (5)	C235—C236—C231	120.6 (5)
C146—C145—H14C	120.6	C235—C236—H23E	119.7
C144—C145—H14C	120.6	C231—C236—H23E	119.7
C143—C144—C145	119.6 (5)	C234—C235—C236	121.0 (5)
C143—C144—H14D	120.2	C234—C235—H23F	119.5
C145—C144—H14D	120.2	C236—C235—H23F	119.5
C135—C136—C131	121.1 (5)	C235—C234—C233	118.9 (5)
C135—C136—H13F	119.5	C235—C234—H23G	120.5
C131—C136—H13F	119.5	C233—C234—H23G	120.5
C135—C134—C133	119.2 (5)	C232—C233—C234	120.8 (5)
C135—C134—H13G	120.4	C232—C233—H23H	119.6
C133—C134—H13G	120.4	C234—C233—H23H	119.6
C136—C135—C134	120.5 (5)	C246—C245—C244	120.4 (5)
C136—C135—H13H	119.8	C246—C245—H24D	119.8
C134—C135—H13H	119.8	C244—C245—H24D	119.8
C144—C143—C142	121.6 (5)	C243—C244—C245	119.5 (5)
C144—C143—H14E	119.2	C243—C244—H24E	120.2
C142—C143—H14E	119.2	C245—C244—H24E	120.2
N13—C14—H14F	109.5	N23—C24—H24F	109.5
N13—C14—H14G	109.5	N23—C24—H24G	109.5
H14F—C14—H14G	109.5	H24F—C24—H24G	109.5
N13—C14—H14H	109.5	N23—C24—H24H	109.5
H14F—C14—H14H	109.5	H24F—C24—H24H	109.5
H14G—C14—H14H	109.5	H24G—C24—H24H	109.5
			
O11—P1—N12—C131	−28.0 (4)	O21—P2—N22—C231	−25.4 (4)
N13—P1—N12—C131	−157.7 (3)	N23—P2—N22—C231	−155.6 (3)
N11—P1—N12—C131	89.6 (4)	N21—P2—N22—C231	91.3 (4)
O11—P1—N12—C13	155.0 (3)	O21—P2—N22—C23	157.6 (3)
N13—P1—N12—C13	25.3 (4)	N23—P2—N22—C23	27.4 (4)
N11—P1—N12—C13	−87.4 (4)	N21—P2—N22—C23	−85.7 (4)
O11—P1—N13—C141	−76.1 (4)	O21—P2—N23—C241	−78.4 (4)
N12—P1—N13—C141	49.2 (4)	N22—P2—N23—C241	47.0 (4)
N11—P1—N13—C141	166.4 (3)	N21—P2—N23—C241	163.9 (3)
O11—P1—N13—C14	100.7 (5)	O21—P2—N23—C24	94.6 (4)
N12—P1—N13—C14	−134.0 (5)	N22—P2—N23—C24	−140.0 (4)
N11—P1—N13—C14	−16.8 (5)	N21—P2—N23—C24	−23.1 (5)
O11—P1—N11—C11	175.3 (4)	O21—P2—N21—C21	174.9 (4)
N13—P1—N11—C11	−58.8 (4)	N23—P2—N21—C21	−58.6 (4)
N12—P1—N11—C11	54.4 (4)	N22—P2—N21—C21	54.6 (4)
C13—N12—C131—C136	−39.0 (6)	C24—N23—C241—C242	−130.4 (5)
P1—N12—C131—C136	143.8 (4)	P2—N23—C241—C242	43.1 (6)
C13—N12—C131—C132	137.3 (4)	C24—N23—C241—C246	51.1 (6)
P1—N12—C131—C132	−39.9 (6)	P2—N23—C241—C246	−135.4 (4)
C14—N13—C141—C142	−131.8 (5)	P2—N21—C21—O22	8.5 (7)
P1—N13—C141—C142	45.3 (5)	P2—N21—C21—C22	−170.9 (3)
C14—N13—C141—C146	49.2 (6)	Cl21—C22—C21—O22	1.8 (6)
P1—N13—C141—C146	−133.7 (4)	Cl23—C22—C21—O22	−119.6 (4)
C146—C141—C142—C143	0.2 (7)	Cl22—C22—C21—O22	121.6 (4)
N13—C141—C142—C143	−178.8 (4)	Cl21—C22—C21—N21	−178.7 (3)
C136—C131—C132—C133	0.6 (7)	Cl23—C22—C21—N21	59.9 (5)
N12—C131—C132—C133	−175.7 (4)	Cl22—C22—C21—N21	−58.9 (5)
P1—N11—C11—O12	3.4 (8)	C242—C241—C246—C245	−1.5 (7)
P1—N11—C11—C12	−175.9 (3)	N23—C241—C246—C245	177.0 (4)
Cl11—C12—C11—O12	−120.4 (5)	C23—N22—C231—C236	−40.6 (6)
Cl12—C12—C11—O12	0.1 (6)	P2—N22—C231—C236	142.1 (4)
Cl13—C12—C11—O12	119.3 (5)	C23—N22—C231—C232	135.6 (4)
Cl11—C12—C11—N11	59.0 (5)	P2—N22—C231—C232	−41.6 (6)
Cl12—C12—C11—N11	179.5 (3)	C236—C231—C232—C233	0.5 (7)
Cl13—C12—C11—N11	−61.3 (5)	N22—C231—C232—C233	−175.8 (4)
C142—C141—C146—C145	−0.6 (7)	C244—C243—C242—C241	−0.2 (7)
N13—C141—C146—C145	178.5 (4)	C246—C241—C242—C243	1.3 (7)
C131—C132—C133—C134	0.2 (7)	N23—C241—C242—C243	−177.2 (4)
C141—C146—C145—C144	0.9 (7)	N22—C231—C236—C235	175.3 (4)
C146—C145—C144—C143	−1.0 (7)	C232—C231—C236—C235	−1.0 (7)
C132—C131—C136—C135	0.0 (7)	C231—C236—C235—C234	0.2 (8)
N12—C131—C136—C135	176.3 (4)	C236—C235—C234—C233	1.2 (8)
C132—C133—C134—C135	−1.6 (8)	C231—C232—C233—C234	0.9 (7)
C131—C136—C135—C134	−1.4 (8)	C235—C234—C233—C232	−1.7 (7)
C133—C134—C135—C136	2.2 (8)	C241—C246—C245—C244	0.7 (8)
C145—C144—C143—C142	0.7 (8)	C242—C243—C244—C245	−0.6 (8)
C141—C142—C143—C144	−0.3 (7)	C246—C245—C244—C243	0.4 (8)

### Hydrogen-bond geometry (Å, °)

**Table:**

*D*—H···*A*	*D*—H	H···*A*	*D*···*A*	*D*—H···*A*
N11—H11B···O21	0.86	1.91	2.741 (5)	161
N21—H21B···O11	0.86	1.93	2.752 (5)	159

## References

[bb1] Amirkhanov, V., Sieler, J., Trush, V., Ovchynnikov, V. & Domasevitch, K. (1997). *Z. Naturforsch. Teil B*, **52**, 1194–1198.

[bb2] Farrugia, L. J. (1997). *J. Appl. Cryst.* **30**, 565.

[bb3] Farrugia, L. J. (1999). *J. Appl. Cryst.* **32**, 837–838.

[bb4] Gholivand, K., Alizadehgan, A., Mojahed, F. & Soleimani, P. (2008*a*). *Polyhedron*, **27**, 1639–1649.

[bb5] Gholivand, K., Vedova, C., Erben, M., Mahzouni, H., Shariatinia, Z. & Amiri, S. (2008*b*). *J. Mol. Struct.* **874**, 178–186.

[bb6] Gubina, K., Ovchynnikov, V., Amirkhanov, V., Sliva, T., Skopenko, V., Głowiak, T. & Kozłowski, H. (1999). *Z. Naturforsch. Teil B*, **54**, 1357–1359.

[bb7] Gubina, K., Ovchynnikov, V., Świątek-Kozłowska, J., Amirkhanov, V., Sliva, T. & Domasevitch, K. (2002). *Polyhedron*, **21**, 963–967.

[bb8] Helm, M., Katz, T., Imioczyk, R., Hands, R. & Norman, A. (1999). *Inorg. Chem.* **38**, 3167–3172.

[bb9] Jaroslav, K. & Swetdloff, F. (1985). US Patent 4 517 003.

[bb10] Katti, K., Pinkerton, A. & Cavell, R. (1991). *Inorg. Chem.* **30**, 2631–2633.

[bb11] Kirsanov, A. & Derkach, G. (1956). *Zh. Obshch. Khim.* **26**, 2009–2014.

[bb12] Oczko, G., Legendziewicz, J., Trush, V. & Amirkhanov, V. (2003). *New J. Chem.* **27**, 948–956.

[bb13] Oxford Diffraction (2006). *CrysAlis CCD* and *CrysAlis RED* Oxford Diffraction Ltd, Abingdon, England.

[bb14] Rebrova, O., Biyushkin, V., Malinovski, T., Ovrucki, V., Procenko, L., Dneprova, T. & Mazus, M. (1982). *Dokl. Akad. Nauk USSR*, **324**, 103–108.

[bb15] Sheldrick, G. M. (2008). *Acta Cryst.* A**64**, 112–122. 10.1107/S0108767307043930 18156677

[bb16] Trush, E., Amirkhanov, V., Ovchynnikov, V., Świątek-Kozłowska, J., Lanikina, K. & Domasevitch, K. (2003). *Polyhedron*, **22**, 1221–1229.

